# Iterative reconstruction improves image quality and reduces radiation dose in trauma protocols; A human cadaver study

**DOI:** 10.1177/20584601211055389

**Published:** 2021-11-18

**Authors:** Johannes Clemens Godt, Cathrine K Johansen, Anne Catrine T Martinsen, Anselm Schulz, Helga M Brøgger, Kristin Jensen, Arne Stray-Pedersen, Johann Baptist Dormagen

**Affiliations:** 1 Department of Radiology and Nuclear Medicine, 56633 Oslo University Hospital Ullevål, Oslo, Norway; 2 Institute of Clinical Medicine, 6305 University of Oslo, Oslo, Norway; 3 The Research Department, Sunnaas Rehabilitation Hospital, Norway; 4 Faculty of Health Sciences, 60499 Oslo Metropolitan University, Oslo, Norway; 5 Department of Diagnostic Physics, 155272 Oslo University Hospital, Oslo, Norway; 6 Department of Forensic Sciences, 155272 Oslo University Hospital, Oslo, Norway

**Keywords:** X-ray, computed tomography, radiation dose, image processing, iterative image reconstruction

## Abstract

**Background:**

Radiation-related cancer risk is an object of concern in CT of trauma patients, as these represent a young population. Different radiation reducing methods, including iterative reconstruction (IR), and spilt bolus techniques have been introduced in the recent years in different large scale trauma centers.

**Purpose:**

To compare image quality in human cadaver exposed to thoracoabdominal computed tomography using IR and standard filtered back-projection (FBP) at different dose levels.

**Material and methods:**

Ten cadavers were scanned at full dose and a dose reduction in CTDIvol of 5 mGy (low dose 1) and 7.5 mGy (low dose 2) on a Siemens Definition Flash 128-slice computed tomography scanner. Low dose images were reconstructed with FBP and Sinogram affirmed iterative reconstruction (SAFIRE) level 2 and 4. Quantitative image quality was analyzed by comparison of contrast-to-noise ratio (CNR) and signal-to-noise ratio (SNR). Qualitative image quality was evaluated by use of visual grading regression (VGR) by four radiologists.

**Results:**

Readers preferred SAFIRE reconstructed images over FBP at a dose reduction of 40% (low dose 1) and 56% (low dose 2), with significant difference in overall impression of image quality. CNR and SNR showed significant improvement for images reconstructed with SAFIRE 2 and 4 compared to FBP at both low dose levels.

**Conclusions:**

Iterative image reconstruction, SAFIRE 2 and 4, resulted in equal or improved image quality at a dose reduction of up to 56% compared to full dose FBP and may be used a strong radiation reduction tool in the young trauma population.

## Introduction

Computed tomography is one of the most important diagnostic medical tools nowadays.^1^ Due to the use of radiation, the hazard from CT is a potentially increased cancer risk, especially in the younger population, including the trauma population with an average age of 35 years.^2–4^ CT is highly accurate for detecting serious trauma-related pathology and crucial for severely injured patients who need a prompt diagnostic evaluation of their injuries.^5^ At our institution, a single-pass split-bolus CT of thorax and abdomen is applied to the majority of trauma patients.^6^ For this type of thoracoabdominal CT scan, data from our National Radiation and Nuclear Safety Authority report an average dose of 14.2 mSv.^7^ In cases of pelvic or urinary tract injury, further arterial or delayed phase scans are obtained and increase radiation dose is administered to the patient.^8,9^ Filtered back-projection has traditionally been the most widely used image reconstruction method, but dose reduction potential of this method is limited.^10,11^ Iterative reconstruction techniques have been introduced, by all CT vendors, to reduce image noise compared to FBP.^12–14^ Sinogram affirmed iterative reconstruction (SAFIRE^®^, Siemens Healthineers, Erlangen, Germany) is decreasing image noise and artefacts, and preserving edges, in a statically iterative process with five different levels of IR levels available where SAFIRE 1 is the mildest and SAFIRE 5 the strongest.^13,15^

Performing repeated CT scans of the same patient for study purpose is not possible due to unnecessary radiation exposure and ethical concerns.^16^ Thus, different IR techniques have been performed as phantom studies.^13,17^ However, human cadaver studies allow multiple scans of the same individual and are superior for presenting anatomy and the clinical reading approach, compared to phantom studies.^18,19^ The purpose of the present human cadaver study was to compare image quality in thoracoabdominal CT reconstructed with SAFIRE IR to standard FBP reconstruction techniques at different dose levels.

## Material and Methods

This study was approved by the Director of Public Prosecutions. Ten human cadavers (median age 33, range 17-90) were anonymously scanned at the Department of Forensic Sciences, Oslo University Hospital, on a scanner used for cadaveric examinations only. Interval between death and CT scanning was maximum 4 days (range 1–4). All CT scans were performed in craniocaudal orientation with arms positioned downward alongside the abdomen. Cadavers with penetrating injury or lacerations were not included. Intravenous contrast material was not applied.

### CT scanning parameters and image acquisition

All CT examinations were performed on a Siemens Somatom Flash 128-slice CT Scanner (Siemens Healthineers, Erlangen, Germany) produced October 2014, installed 13.11.2014. Regular service was performed once a year. Flying focal spot collimation was 128 × 0.6 mm. Scans were performed with Caredose automatic exposure control using reference mAs fixed at 200 for FD, 120 kVp, gantry rotation time 0.5 s, and a pitch of 0.9.

All cadavers were scanned from superior thoracic aperture to lesser trochanter without gantry tilt at three different levels of radiation dose: full dose (FD, mean CTDIvol = 15.2 mGy), dose reduction by 5 mGy (LD1, mean CTDIvol = 9.1 mGy), and 7.5 mGy (LD2, mean CTDIvol = 6.6 mGy). The scan field of view was adjusted to patient size. Mean displayed field of view was 41.1 cm (range 33–48 cm). 3 mm axial, sagittal, and coronal reformations were reconstructed. Images were reconstructed with FBP reconstruction (kernel B31 F) and SAFIRE (kernel I31 F) IR algorithm at level 2 and 4.

In total, seven CT image series of thorax and abdomen were evaluated per each human cadaver: FD FBP, and for both LD1 and LD2 images series reconstructions in FBP, SAFIRE 2 and 4. Radiation dose for FD, LD1, and LD2 image series was calculated using the dose length product (DLP)—conversion factor method, where the effective dose E = DLP *anatomic area conversion factor.^7,20^

### Image quality analysis

Image evaluation was performed on a PACS workstation (Syngo Via version VB20, Siemens, Munich, Germany). Each cadaver, dose level, and reconstruction type were assigned a unique ID code during the scanning and reconstruction process. Thus, all CT images analyzed in this study were anonymized.

Quantitative image quality analysis was performed by the main investigator of the study. A circular region of interest (ROI) of 0.5 cm^2^ size was manually placed in liver and spleen parenchyma avoiding vascular structures, psoas muscle, paravertebral fat tissue and air. Mean and standard deviation in Hounsfield Units (HU) were recorded within each ROI. Contrast-to-noise ratio between liver and spleen was computed by the following formula

$$\text{CNR}=\frac{2{\left({\text{s}}_{2}-{\text{s}}_{1}\right)}^{2}}{{\text{σ}}_{1}^{2}+{\text{σ}}_{2}^{2}}$$

where s denotes the signal (mean CT number) and σ the standard deviation (noise), and subscripts 1 and 2 represent the two target ROIs (liver parenchyma and spleen, respectively).^21^ Signal-to-noise ratio was calculated as

$$\text{SNR}=\frac{\text{s}}{\text{σ}}$$

for each ROI.

Qualitative image quality assessment was performed independently by four board-certified radiologists with more than 10 years of experience. A training session was performed before evaluation start, to familiarize the readers with the assessment methodology. A total number of 70 thoracoabdominal CT image sets were evaluated in randomized order over several days for different high and low contrast anatomical structures. Readers were blinded to radiation dose level and reconstruction techniques applied. All image evaluation was performed on axial slices.

Scores from 1 (worst) to 5 (best) were given for evaluation of visually sharp reproduction of 10 anatomical structures (Table 1) adapted and slightly modified from the European guidelines for image quality in abdominal CT.^22^ All evaluation was done avoiding areas of major pathologic or postmortem changes. In addition, readers gave scores for the overall impression of image quality, image noise, image contrast, plastic look of images and internal artifacts. Plastic look was defined as artificial or plastic-like appearance of structures seen in CT images reconstructed with IR.^23,24^

**Table 1.** Overview of items for qualitative image evaluation.

Visually sharp reproduction of:	Evaluation window level	Evaluation scale
Lung septae	Lung window	1 = non-diagnostic image quality 2 = severe blurring with poorly defined structures 3 = moderate blurring with restricted evaluation 4 = slight blurring with unrestricted image evaluation possible 5 = excellent image quality
Lung bronchiae		
Lung fissure		
Second costa, right side	Bone window	
Aortic arch	Abdominal window	
Crus diaphragm		
Liver		
Spleen		
Kidneys		
Rectum		
Overall impression of:	Abdominal window	1 = unacceptable 2 = suboptimal 3 = satisfactory 4 = good 5 = excellent
Image quality		
Noise		
Image contrast		
Plastic look		
Internal artifacts		

### Statistical analyses

Visual grading regression analysis of the qualitative image assessment was performed with STATA SE 14 (StataCorp, College Station, TX) using the command meologit.^25,26^ Reader was specified as a random effect and reconstruction method as fixed effect. A z-test was used to determine statistical significance; the level of significance was set at 0.05. Wilcoxon test was used for the evaluation of radiation dose and quantitative image quality. Interobserver agreement was evaluated with intraclass correlation coefficient (ICC) using SPSS version 25 (SPSS Inc., Chicago, IL, USA). ICC <0.5 was defined as poor reliability, 0.51–0.75 as moderate, 0.76–0.9 as good, and 0.90–1.00 as excellent reliability.^27^

## Results

Radiation dose reduction was 40% for the LD1 and 56% for the LD2 (see Table 2). An overview of CT scanning parameters and the corresponding CTDIvol and DLP of all full dose scans is given in Table 3 for each cadaver.

**Table 2.** Radiation dose results.

CTDIvol (mGy), mean (range)	15.2 (9.7 to 19.4)	9.1 (4.2 to 12.3)	6.6 (1.9 to 10.2)
DLP (mGy cm), mean (range)	1162.7 (676.0 to 1662.0)	698.0 (291.0 to 10540)	509.6 (136.0 to 878.0)
Radiation dosage, mSV, mean (range)	17.4 (10.1 to 24.9)	10.5 (4.4 to 15.8)	7.6 (2.0 to 13.1)
% dosage reduction from FD	not applicable	39.7%* (p=0.005)	56.3%* (p=0.005)

*Statistical significance.

FD: full dose; LD1/2: low-dose level 1/2.

**Table 3.** CT scanning and corresponding radiation dose parameters, full dose CT.

Cadaver	kV	mAs (eff.)	Slice thickness	CTDIVol (mGy)	DLP (mGy cm)
1	120	238	3 mm	16,11	1175
2		243		16,48	1250
3		182		12,33	1054
4		235		15,91	1201
5		266		18,02	1360
6		142		9,66	676
7		229		15,51	1236
8		214		14,47	1079
9		192		13,04	968
10		200		13,57	1020

kV: kilovolt; mAs (eff.): milli Ampere seconds (effective); DLP: dose length product.

### Quantitative image evaluation

Results from quantitative image evaluation are shown in Table 4. No significant difference in HU measurements was seen, regardless of the dose level or IR method applied. For LD1 and LD2-scans, CNR liver/spleen and SNR values of liver, spleen, psoas, fat and air were significantly higher for the images reconstructed with IR technique compared to the FBP reconstructed images, with exception of SNR for fat at SAFIRE 2-level. CNR measurements for LD1 images showed an improvement of 76% for SAFIRE 2 and 258% for SAFIRE 4 compared to LD1 FBP (*p* = 0.005). For LD2 images, CNR increased 64% for SAFIRE 2 and 238% for SAFIRE 4 images compared to LD2 FBP (*p* = 0.021/0.005).

**Table 4.** Quantitative image quality evaluation results, Hounsfield Units (HU). All values presented as mean (range).

HU Results Mean (Range)	Full dose FBP	LD1 FBP	LD1 SAF2	LD1 SAF4	LD2 FBP	LD2 SAF2	LD2 SAF4
Liver	62 (48 to 72)	62.1 (48 to 73)	61.9 (48 to 73)	61.8 (48 to 73)	60.7 (45 to 72)	61.2 (48 to 72)	61.2 (48 to 71)
Spleen	50.9 (31 to 65)	51.1 (30 to 73)	50.8 (29 to 73)	50.8 (29 to 73)	50.2 (30 to 78)	50.1 (30 to 78)	49.9 (30 to 78)
Psoas	52.2 (35 to 62)	52.1 (35 to 65)	51.6 (34 to 64)	51.5 (33 to 64)	51 (38 to 64)	50.8 (36 to 64)	50.5 (36 to 64)
Fat	−77 (−102 to −26)	−75.5 (−97 to −38)	−75.5 (−90 to −38)	−75.2 (−96 to −37)	−75.4 (−97 to −32)	−75.8 (−102 to − 31)	−75.3 (−103 to −31)
Air	−969.8 (−982 to −976)	−968.7 (−988 to −937)	−968.6 (−987 to −938)	−986.5 (−987 to −938)	−966.2 (−986 to −921)	−966.9 (−985 to −921)	−966.2 (−984 to −921)
CNR L/S	2.9 (0.4 to 11.3)	1.7 (0.2 to 5.4)	*3 (0.6* to *9.5)*	*6,1 (0.85* to *18)*	1.3 (0.0 to 5.5)	2.2 (0.0 to 9.8)	4.4 (0.1 to 18.3)
SNR Liver	6.8 (2.5 to 11.7)	5.1 (1.8 to 10.4)	*6.9 (3.33* to *14.6)*	*10.4 (4.7* to *24.3)*	4 (1,8 to 6.3)	5 (1.6 to 7.6)	7.2 (3.2 to 11.5)
SNR Spleen	4.9 (1.7 to 10.5)	3.5 (1.6 to 6)	*4.4 (2.1* to *7.2)*	*6.2 (2.9* to *10.8)*	3.2 (1.8 to 6.2)	4.2 (1.8 to 8.5)	5.8 (2.5 to 11.1)
SNR Psoas	4.6 (2.9 to 6.2)	4 (1.8 to 7.1)	*5.3 (2.3* to *9.3)*	*7 (3.3* to *11.2)*	3.4 (1.4 to 6.2)	4.2 (2 to 6.9)	6.3 (2.4 to 11)
SNR Fat	−6.6 (−11.4 to −2.4)	−5.6 (−9.7 to −9.2)	−*6.7 (*−*12.4 to* −*3.4)*	−*9.5 (*−*17.4 to* −*4.6)*	−5.4 (−8.8 to −1.7)	−6.2 (−10.6 to −2.1 )	−8.7 (−17 to −2.8)
SNR Air	−88.4 (−243.7 to −75.1)	−95.4 (−161.8 to −23.4)	−*126.2 (*−*195.2 to* −*24.1)*	−*161.3 (*−*246.7 to* −*24.7)*	−73.8 (−121 to 14.7)	−93.9 (−161.7 to −14.4)	−127.2 (−242.5 to −14.6)

*italic cell values = significant diffference compared to FBP at same dosage level* CNR L/S: Contrast- to -noise ratio Liver/Spleen; SNR: Signal- to -noise ratio; FD FBP: full dose filtered-back projection; LD1/2: low dose level 1/2; SAF: SAFIRE.

### Qualitative image evaluation

Both reduced dose LD1 and LD2 FBP images were scored inferiorly compared to FD FBP images (Table 5). Noticeably, readers concluded with higher ratings for LD1-images at SAFIRE 4-level compared to FD FBP for all items despite of lunge septae. For LD2 images at the same comparison, only lunge septae, rectum, kidney and impression of plastic look were scored better at FD FBP. For all comparisons at the same level of low dose, both SAFIRE 2 and SAFIRE 4 images were scored better than FBP images, with exception of overall impression of plastic look. For several criteria, such as liver, spleen, kidney, and overall impression of image quality, results were significantly different in favor of images reconstructed with SAFIRE 2 or 4.

**Table 5.** Qualitative image quality evaluation results.

1	FD FBP	FD FBP	FD FBP	FD FBP	FD FBP	FD FBP	LD1 FBP	LD1 FBP	LD2 FBP	LD2 FBP
2	LD1 FBP	LD2 FBP	LD1 SAF2	LD2 SAF2	LD1 SAF4	LD2 SAF4	LD1 SAF 2	LD1 SAF 4	LD2 SAF2	LD2 SAF4
Lung septae	*	*					*			*
Lung bronchiae	*	*	†	†	*	*	†	†	*	*
Lung fissure	†	†	†	†				*	†	†
Costa 2, right side							*	*		*
Aortic arch	†	†		*			*	*	†	†
Crus diaphragm	*	*					*	*	†	†
Liver	*	*		*			*	*	*	*
Spleen	*	*					*	*	*	*
Kidney	*	*	*	*			*	*	*	*
Rectum	*	*	*	*				*	*	*
Overall impression of image quality	*	*		*	*		*	*	*	*
Overall impression of noise	*	*		*	*	*	*	*	*	*
Overall impression of image contrast	*	*			*		*	*		*
Overall impression of plastic look	*	*			†	*	*	*	*	*
Overall impression of internal artifacts					*			*		*

White table cells = 1 better (* = significant) gray shaded table cells = 2 better (* = significant).

†Visual grading regression analysis results did not converge sufficient to perform a calculation.

FD FBP: full dose filtered-back projection; LD1/2: low dose level 1/2; SAF: SAFIRE.

Only in 16 of 150 comparisons (10.7%), VGR analysis data did not converge and a calculation was not possible. This occurred only in evaluation of lung fissure, lung septae, aortic arch, crus diaphragm, and plastic look.

**Figure 1.**
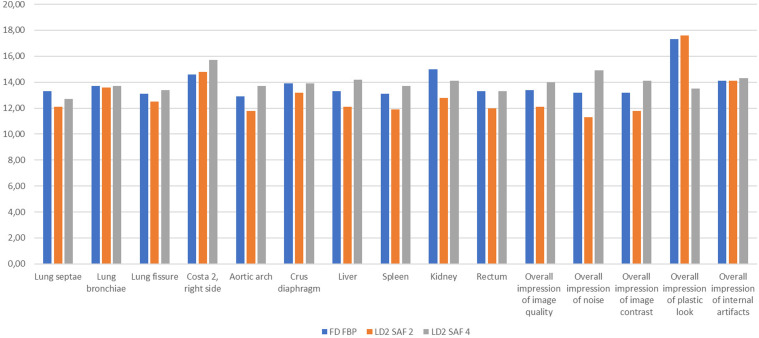
Visual grading regression analysis. Total score per radiologist for each criteria, average for all patients. FD FBP: Full-dose filtered-back projection; LD2: low dose level 2; SAF: SAFIRE.

Figure 1 shows the sum of the total score per radiologist for each criteria, displayed as average for all patients. In this diagram, LD2 scans at SAFIRE 2 and 4 reconstruction was compared to FD FBP. Noteworthy, LD2 SAFIRE 4 images had an equal or a higher score sum than FD FBP for all image evaluation criteria except for lung septae, kidney, and plastic look. In addition, overall impression of plastic look was scored best in the LD2 SAFIRE 2 image series.

Interobserver agreement results for each item and image series are presented in Table 6. An inter-reader reliability over 0.5 was noted in 82 of 105 assessments, resulting in moderate, good, or excellent inter-reader agreement in 78% of all evaluations. Highest ICC level was noted for evaluation of rectum in LD 2 SAFIRE 4 images (0.929). Noticeably, mean ICC for SAFIRE 4 images was superior to FD FBP at both low dose levels.

**Table 6.** Interobserver agreement results for qualitative image evaluation.

ICC (Mean)	FD FBP	LD 1 FBP	LD1 SAF2	LD1 SAF4	LD2 FBP	LD2 SAF2	LD2 SAF4
Lung septae	0.71	0.88	0.68	0.62	0.20	0.78	0.81
Lung bronchiae	0.81	0.85	0.79	0.91	0.64	0.87	0.86
Lung fissure	0.82	0.76	0.81	0.80	0.82	0.88	0.79
Costa 2, right side	0.24	0.56	0.41	0.57	0.00	0.26	0.08
Aortic arch	0.77	0.88	0.86	0.81	0.88	0.65	0.85
Crus diaphragm	0.70	0.86	0.83	0.79	0.82	0.76	0.81
Liver	0.39	0.73	0.59	0.61	0.64	0.48	0.77
Spleen	0.44	0.75	0.48	0.75	0.72	0.62	0.75
Kidney	0.73	0.45	0.81	0.64	0.76	0.74	0.89
Rectum	0.70	0.80	0.81	0.76	0.80	0.87	0.93
Overall impression of image quality	0.46	0.85	0.70	0.68	0.70	0.47	0.76
Overall impression of noise	0.39	0.55	0.42	0.64	0.58	0.71	0.77
Overall impression of image contrast	0.51	0.85	0.83	0.41	0.61	0.63	0.74
Overall impression of plastic look	0.61	0.77	0.07	−0.40	0.61	−0.58	−0.13
Overall impression of internal artifacts	0.41	0.53	0.20	0.41	−0.30	−0.46	0.30
Mean	0.56	0.73	0.60	0,.56	0.61	0.44	0.68

FD FBP: full dose filtered-back projection; LD1/2: low dose level 1/2; SAF: SAFIRE.

## Discussion

In CT diagnostics, the examinations should be optimized with respect to image quality and radiation dose according to the ALARA and ALARP principles of radiation protection.^28^ This is specially of concern in emergency radiology, where long scans on a young patient population demand an optimal balance of radiation dose and image quality.^29,30^

Vendors have developed new technologies and reconstruction techniques to improve image quality and reduce radiation dose, like IR techniques. Several studies have been performed to evaluate new technologies.^13,31–33^ Still, there has been a lack of a systematic assessment of quantitative and qualitative image quality and radiation dose on humans for different dose levels and reconstruction techniques in emergency CT.

This study showed that readers preferred images reconstructed with IR techniques (SAFIRE 2 and 4) at a dose reduction of 40 and 56%, compared to the full dose FBP images. Significant improvement of CNR values was seen in low dose images reconstructed with SAFIRE. Interobserver agreement was superior for low dose images reconstructed with SAFIRE 4 compared to FD FBP.

The image interpretation setting was designed as similar as possible to an everyday clinical routine setting. Therefore, readers were presented a whole axial CT stack of thorax and abdomen on the same PACS workstation normally used in daily routine.

In our knowledge, only two other studies on SAFIRE IR techniques have been performed on human cadavers, both evaluated CT of the chest only. Macri et al. performed a study on 18 cadavers at a low dose level of 40 mAs and ultra-low-dose level of 10 mAs compared to full dose CT at 200 mAs. Dose reduction was 80 and 95%, compared to 39 and 56% in our study. SAFIRE 3, 4, and 5 was applied on the image series with reduced dose and led to improvement of CNR and SNR. Subjective image quality on a five-point Likert scale was rated excellent/good for both FBP and low dose images, and good/fair for the ultra-low-dose images.^16^

DeCrop et al. evaluated a series of low dose CT reconstructed with SAFIRE 1, 3, and 5 level on both Catphan phantom and Thiel embalmed cadavers. Their findings are of importance, since they emphasize the need of cadaver studies in addition to phantom studies. Standard reference mAs was 90; several scans were evaluated at reference mAs values between 12 and 150. Subjective cadaver image quality was evaluated by use of visual grading analysis. Quantitative image quality analysis was performed only for the phantom evaluation, not for the human cadavers. Potential dose reduction showed to be lower when based on clinical image quality evaluation in cadavers (27–37.4%) compared to image quality evaluation based on objective parameters in a phantom (14–71.5%).^34^

Mueck et al. performed a study of contrast-enhanced postmortem chest CT. They compared a series of full dose images reconstructed with ASIR (Adaptive statistic IR) with 5 different levels of low dose CT. All low dose images were reconstructed with MBIR (Model-based IR) and qualitative image improvement compared to full dose ASIR images was shown down to a level of 75% dose reduction.^35^

In the literature, there are no cadaver studies evaluating SAFIRE in the abdomen. Only one cadaver study of IR in abdominal CT has been published so far. Moloney et al.^36^ evaluated ASIR and MBIR in five cadavers at full dose and three low dose levels. SNR was significantly higher in MBIR image series, compared to FBP and ASIR images. Image quality was scored significantly better for MBIR images compared to ASIR and FBP, but not for ASIR compared to FBP images.

Reconstruction methods may influence the HU values, as shown by Jensen et al.^37^ This was not the case in our study, nor in the study of Mueck et al. when comparing MBIR and ASIR in the thorax.

Noise reduction properties of iterative techniques potentially improve SNR and CNR values, demonstrated in previous phantom studies.^13,17^ We could confirm these findings in human cadavers. For the abdomen, Moloney et al.^36^ found, concordant with our results, significantly higher SNR in MBIR image series compared to FBP, even if a different cadaver preservation method was used. Regarding the thorax, Macri et al. did not observe CNR or SNR values superior to reference full dose FBP images for all levels of SAFIRE.^16^ This occurred only in some measurements in our study, such as for SAFIRE 2 at LD2 in muscular tissue. A possible explanation could be the higher dose reduction applied by Macri et al. (up to 95%) compared to our study (up to 56%).

**Figure 2.**
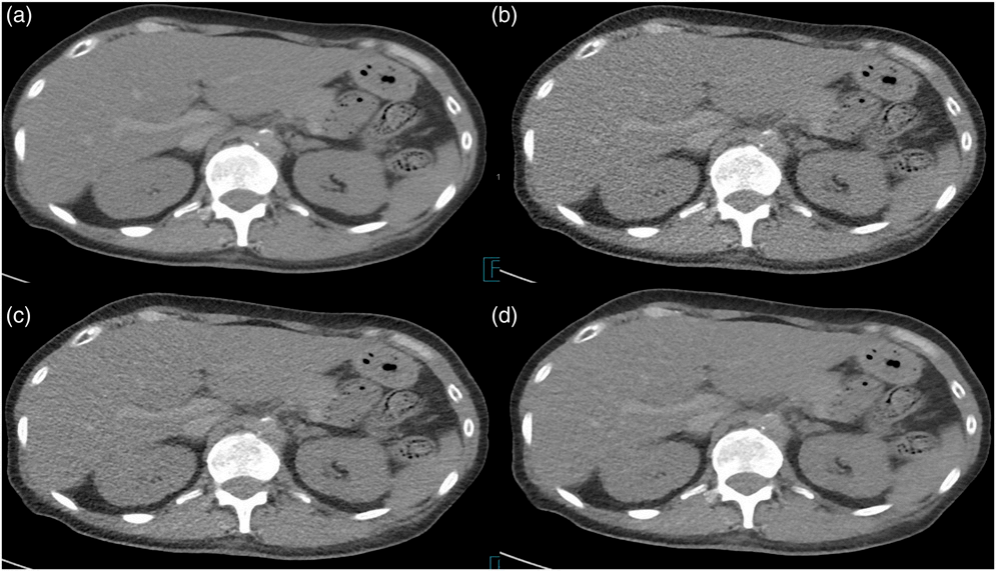
Images of the upper abdomen. Full-dose FBP (a) and three images at low-dose level 2 (LD2). FBP (b), SAFIRE 2 (c), and SAFIRE 4 (d).

**Figure 3.**
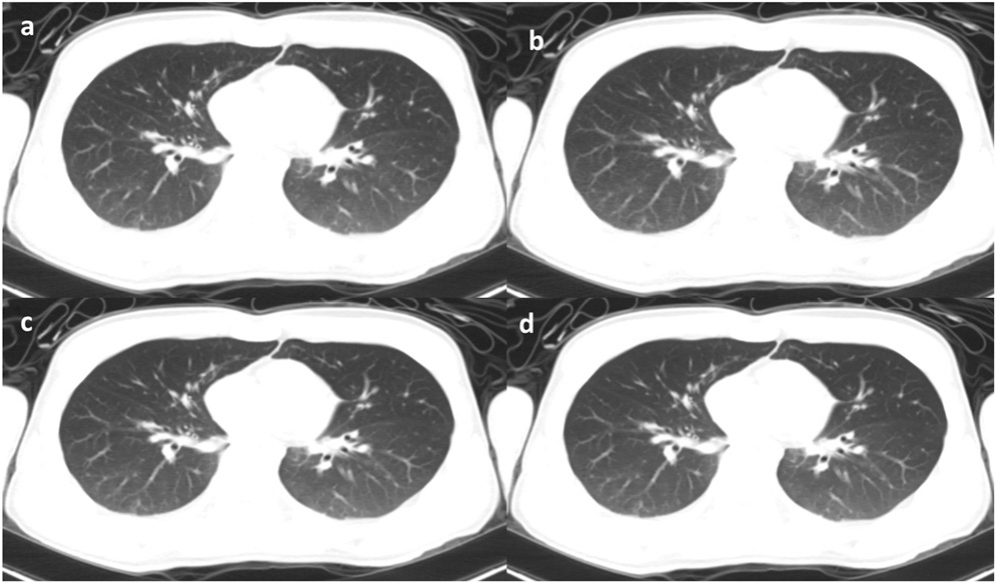
Images of the lung. Full-dose FBP (a) and three images at low-dose level 1 (LD1). FBP (b), SAFIRE 2 (c), and SAFIRE 4 (d).

As stated by DeCrop et al.^34^ caution is recommended when evaluating image quality based on physical-technical parameters like CNR only, also in cadavers. It is of importance that all image evaluation in our study was performed blinded to reconstruction method applied, since there has been reported some skepticism among radiologists in using higher strengths of IR.^38^ In this study, readers preferred SAFIRE 4 reconstructed images for almost all scored items compared to FBP reconstructed images. This was the case both at the same low dose level and when comparing low dose to full dose FBP. A comparison of full dose FBP and LD2 images of the upper abdomen is shown in Figure 2, and images comparing full dose FBP and LD1 of the lung in Figure 3. On the other hand, when comparing SAFIRE 2 low dose images to full dose FBP, readers still favored full dose FBP images for many structures, also for overall impression of image quality. These results confirm that SAFIRE has a better potential of image quality improvement at a higher level applied. This was also supported by the ICC results, where ICC for SAFIRE 4 images was superior to FD FBP at both low dose levels, showing higher reader agreement, Low dose FBP images without IR were not surprisingly scored inferior to full dose FBP images, for most of the items significantly. The only exception was the evaluation of plastic look.

**Figure 4.**
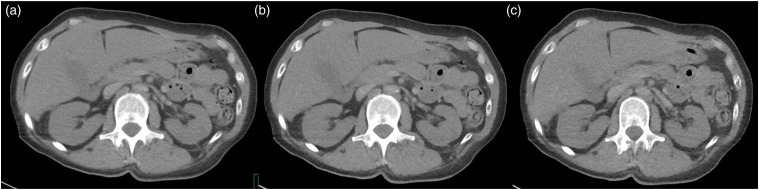
Images of the upper abdomen for comparison of “plastic look” appearance. Full dose FBP (a), LD 1 SAFIRE 4 (b), and LD 2 SAFIRE 4 (c).

“Plastic look” or blotchy appearance can occur in images reconstructed with SAFIRE or other iterative image reconstruction techniques.^39^ This was also the case in our study, and readers experienced SAFIRE 2 and SAFIRE 4 images as having more “plastic look” appearance than in FBP images at different dose levels (Figure 4). This is of clinical importance, since blotchy image appearance can influence detection of small pathological structure changes.^13,32^ Noteworthy, scoring for “plastic look” appearance differed also between FBP images without any IR at different dose levels, with low dose FBP images rated as having less “plastic look”- appearance than full dose FBP images.

When evaluating subjective image quality, an advantage of VGR analysis is that this method can be applied controlling for dependencies between readers and reconstruction methods.^38^ A non-convergent VGR analysis is a consequence of too widespread rating results. In our study, VGR analysis did not converge in some evaluations, all of these situated in the thoracical region. This has probably several reasons: Firstly, the whole spectra of CT density from air to cortical bone is present in this region. Secondly, for items like lung fissure and bronchi, one reason could be that image contrast changes after application of IR, and raters often prefer the image texture they are used to in their daily practice.^40^

In contrast to the chest CT cadaver study of Macri et al., our interobserver agreement results were stable for most of evaluated items also at lower dose images reconstructed with SAFIRE. Variation in grading of image quality is not unusual among radiologists, and our ICC results are comparable even to other in-vivo studies.^38^

Our study has some limitations. Human cadaver studies do not reflect a clinical imaging situation due to lack of pathology, respiratory movements, intravenous contrast media among others. Postmortem changes can occur in the tissue of human cadavers. The impact of intravenous contrast material was not evaluated in our analysis, since absence of ongoing blood circulation in human cadavers requires a complex procedure of contrast-enhanced postmortem CT.^18^ This study focused on image quality and assessment of anatomical structures, not the identification of pathology. Meanwhile, we consider the use of human cadavers instead of a phantom, our prospective study design, and the relatively high number of observation scores as strengths of our study. Phantom studies are easier to perform, but human cadavers resemble closely natural anatomical structures.^40^

In conclusion, application of SAFIRE IR algorithms resulted in equal or improved quantitative and qualitative image quality at lower dose levels compared to FBP images. A considerable amount of dose reduction may be achievable when applying SAFIRE, which will especially gain the younger population of trauma patients.
