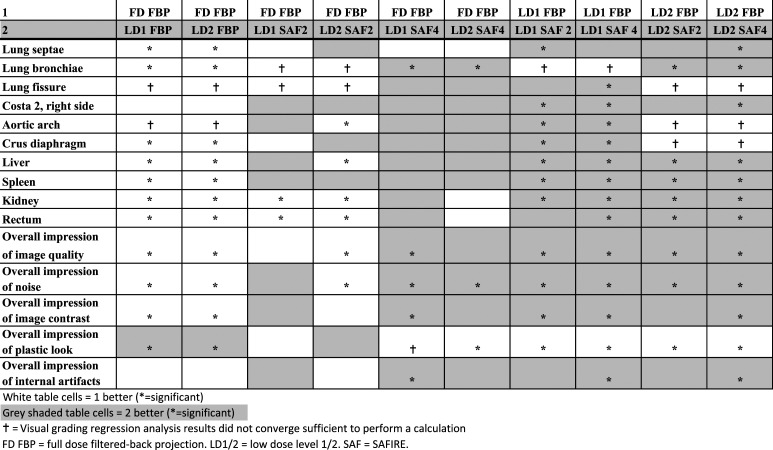# Erratum to “Iterative reconstruction improves image quality and reduces radiation dose in trauma protocols; A human cadaver study”

**DOI:** 10.1177/20584601221081284

**Published:** 2022-05-28

Godt JC, Johansen CT, Martinsen ACT, et al. Iterative reconstruction improves image quality and reduces radiation dose in trauma protocols; A human cadaver study. *Acta Radiol Open* 2021;10(10). doi:10.1177/20584601211055389

In the above-referenced article, the term “spilt bolus” in the Background section of the Abstract should be “split bolus”.

In addition, Table 5 was formatted incorrectly by the Publisher. An updated version of Table 5 with correct shading is provided below.

**Table 5.** Qualitative image quality evaluation results.